# Impact of Cadmium Exposure during Pregnancy on Hepatic Glucocorticoid Receptor Methylation and Expression in Rat Fetus

**DOI:** 10.1371/journal.pone.0044139

**Published:** 2012-09-05

**Authors:** Paula Castillo, Freddy Ibáñez, Angélica Guajardo, Miguel N. Llanos, Ana M. Ronco

**Affiliations:** Laboratory of Nutrition and Metabolic Regulation, Institute of Nutrition and Food Technology (INTA), University of Chile, Santiago, Chile; Shantou University Medical College, China

## Abstract

Adverse fetal environment due to maternal undernutrition or exposure to environmental chemicals alters glucocorticoid (GC) metabolism increasing the risk of metabolic disorders in adulthood. In this study, we investigated the effects of maternal exposure to cadmium (Cd, 50 ppm) during pregnancy in the methylation of fetal hepatic glucocorticoid receptor promoter (GR) and the correlation with its expression and that of the DNA methyltransferases (DNMT1a and 3a). We also studied the expression of liver phosphoenolpyruvate carboxykinase (PEPCK) and acyl-CoA oxidase (AOX), two enzymes involved in the metabolism of carbohydrates and lipids respectively. The methylation of the rat GR gene exon 1_10_ (GR1_10_) in nucleotides -2536 to -2361 was analyzed by pyrosequencing. Quantitative real time PCR was used to assess hepatic GR, PEPCK and AOX mRNA, and their protein levels using Western blotting analysis. Differential methylation was noted across groups at all CpG sites in the GR exon 1_10_ in a sex-dependent manner. In males, CpG were more methylated than the controls (185±21%, p<0.001) but only CpG sites 1,6,7 and 9 showed a significantly different extent of methylation. In addition, a lower expression of GR (mRNA and protein) was found. On the contrary, in females, CpG were less methylated than the controls (62±11%, p<0.05) and overexpressed, affecting PEPCK and AOX expression, which did not change in males. The GR methylation profile correlates with DNMT3a expression which may explain epigenetic sex-dependent changes on GR1_10_ promoter induced by Cd treatment. In conclusion, Cd exposure during pregnancy affects fetal liver DNMT3a resulting in sex-dependent changes in methylation and expression of GR1_10_. Although these effects do not seem to be directly involved in the low birth weight and height, they may have relevant implications for long-term health.

## Introduction

The relevance of prenatal environment on developmental plasticity and phenotypic outcome of the offspring are becoming increasingly acknowledged [1]. Particular attention has recently focused on the potential consequences of epigenetic processes in developmental programming and its role in the susceptibility to chronic diseases in adulthood [2]–[4].

Maternal undernutrition, stress and exposure to toxicants during gestation are among the known factors that alter fetal environment [5]–[7]. One of the mechanisms involved in their adverse effects upon the developing fetus has been associated to alterations in the glucocorticoid (GC) system. In this respect, a prenatal overexposure to GC, either directly or indirectly may induce deleterious early-life events altering birth weight and increasing subsequent development of the common cardiovascular and metabolic disorders in adult life [8]–[10].

We and others have previously reported that cadmium (Cd), a ubiquitously distributed environmental toxicant and main component of tobacco smoke, accumulates in the placenta of pregnant rats inducing low birth weight in high correlation with placental levels of this heavy metal [7], [11], [12]. We have reported that Cd exposure during pregnancy increases levels of circulating corticosterone – the main active GC of rodents – in mothers and offspring, suggesting an involvement of the GC system in some toxic effects induced by Cd [10], [13]. This steroidogenic hormone is regulated by the 11β-hydroxysteroid dehydrogenase enzymes (11β-HSD; isoforms 1 and 2) and mediated by intracellular glucocorticoid receptor (GR) [9].

Despite the clear physiological importance of endogenous GC during development, there are evidence supporting that an excess of exogenous GC during pregnancy is correlated with reduced birth weight and subsequent development of the common cardiovascular and metabolic disorders of adult life, notably hypertension, insulin resistance and type 2 diabetes [9], [14], [15]. Adverse effects of GC exposure could be related to GR, a member of the nuclear hormone receptor superfamily of ligand-activated transcription factors, which are expressed in most fetal tissues from early embryonic stages [16]. In rat, several GR mRNA transcripts have been detected; its expression is tissue-specific and complexly regulated [17]. Eleven exon 1 sequences have been identified and characterized, being transcripts containing exon 1_10_ (GR1_10_) the most predominantly expressed in rat liver (more than 75%). In addition, the expression of this exon is able to change epigenetically by perinatal environmental manipulations, like maternal diet [18]. Prenatal GC treatment, maternal malnutrition and protein-restricted feeding protocols during pregnancy have induced increased, hepatic GR expression in the offspring [18]–[21]. Glucocorticoids regulate several important hepatic processes related with carbohydrate and fat metabolism. For example, hepatic phosphoenolpyruvic-carboxykinase (PEPCK), a rate-limiting step in gluconeogenesis and acyl-CoA oxidase (AOX), a PPARα target gene involved in peroxisomal fatty acid β-oxidation, may be altered directly or indirectly by GC overexposure [22], [23].

It has been demonstrated that placental cells incubated with Cd show lower levels of 11β-HSD2 expression, suggesting that the Cd content in tobacco smoke may induce fetal overexposure to GC and, consequently, low birth weight [24].

In addition, as previously demonstrated in cultured liver cells, we have recently shown that Cd added to cultured choriocarcinoma cells (JEG-3) induced DNA methylation of the 11β-HSD2 gene (*Hsd11b2*), probably through alterations in DNA methyltransferase (DNMT) activity and expression [25], [26]. GR expression has been associated to GR promoter methylation and its regulation has been linked to fetal programming induced by stress or undernutrition [15], [27], [28].

Currently, it is not known whether developmental exposure to Cd alters the methylation of specific or all CpG dinucleotides within gene promoter regions in liver GR1_10_ of rat offspring, and consequently, the expression of enzymes involved in carbohydrates and lipids metabolism. In this study, we exposed pregnant rats to Cd (50 ppm) and examined the effects on individual CpG dinucleotide in the promoter region of fetal hepatic GR gene. We also assessed the relation of CpG methylation with DNMTs (DNMT1a and DNMT3a), its substrate S-adenosyl-methionine (SAM) and the enzymes PEPCK and AOX. Epigenetic mechanisms may have a gender component [29]–[31], and because sex-dependent effects of Cd and GC have also been reported, we evaluated Cd effects separately on female and male offspring.

## Results

### Fetal Characteristics

Fetuses of Cd exposed female rats registered lower birth weight (3.3 g ±0.4 vs 2.9 g ±0.4, mean ±SD, *p<*0.001) and size: 3.8 cm (3.6–3.9) vs 3.6 cm (3.4–3.8) (median p^25^–p^75^, *p<*0.01) compared to controls. When analyzed by sex, this trend continues being female’s weight and size lower than in males. Placentás weights did not change between both groups as litter size, but offsprinǵs livers from exposed mothers weighed less than controls: 0.28 g (0.26–0.30) vs 0.18 g (0.16–0.2), (median p25–p75, *p<*0.001).

### Expression of GR (mRNA and Protein) in Fetal Liver

**Figure 1:**
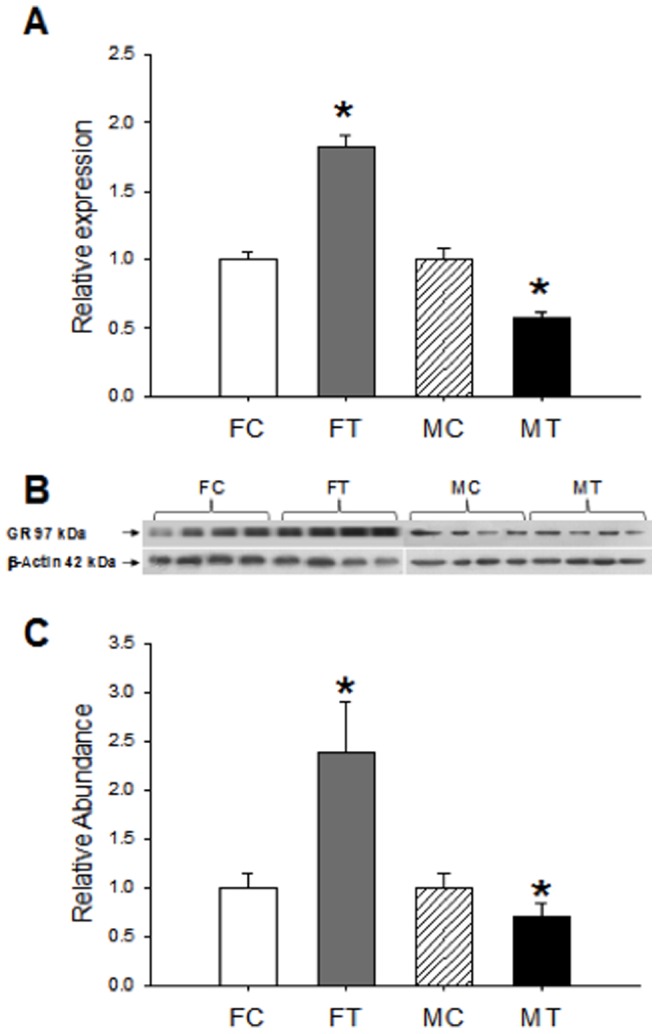
Expression of GR in fetal liver. GR mRNA was determined by real time PCR as described in Material and Methods (A). Results represent the GR/β actin ratio of duplicate experimental determination of 6 different biological samples of female control (FC) and Cd-treated offspring (FT) and male control (MC) and Cd-treated offspring (MT). Western blot analysis for GR protein expression in the liver of control (FC) and treated female (FT) and control (MC) and treated male (MT) fetus (B). GR western blot signals were determined by densitometry and expressed as abundance ratios of GR related to actin (C); *p<0.05 compared with respective control.

Because GR gene transcription is, in part, regulated by early-life environmental events that program tissue GR levels, we examined the expression of GR (mRNA and protein) in liver fetuses of males and females of Cd-treated and control dams. Results described in Fig. 1 show that hepatic GR mRNA levels of treated female fetuses (FT) are higher compared to GR mRNA of control non-exposed females (FC, Fig. 1A). On the contrary, GR mRNA levels of treated male fetuses (MT) are lower than the corresponding control males (MC, Fig. 1A). The GR protein as assessed by WB shows a concordant behavior with GR mRNA (Fig. 1 B and C): it is higher in liver of treated females (FT vs FC) and lower in treated males when comparing to respective controls (MT vs MC, Fig. 1 B and C).

### Methylation at CpG Sites of the Fetal Liver GR1_10_

**Figure 2:**
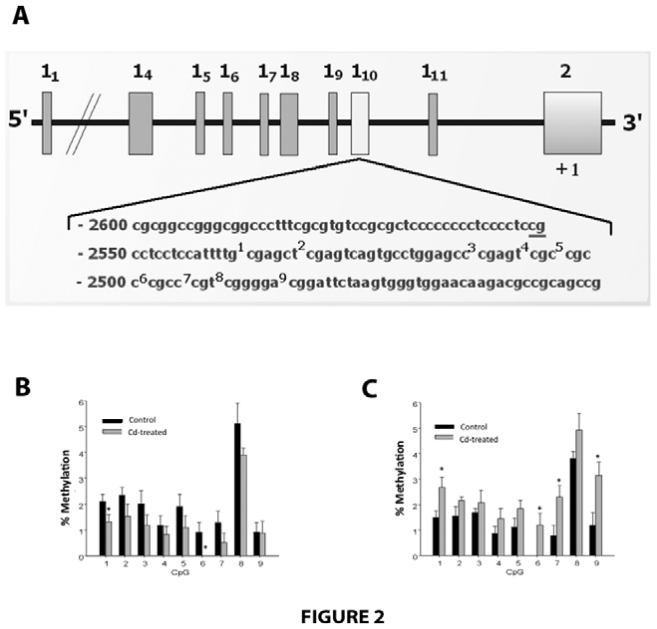
Relative position of alternative exons 1 of the rat GR gene and the CpG- rich region analyzed by bisulphite pyrosequencing at the exon 1_10_. The diagram is based on the previously characterized 5′-end of the rat GR gene, which contains multiple first exons. The numbering is relative to the translational start site (+1), which is located at exon 2 (A). Extent of methylation of the 9 CpG sites in the GR1_10_ exon promoter region in fetal liver of female (B) and male (C) offspring of control and Cd-treated pregnant rats. Results were calculated by averaging the methylation level of each CpG dincleotide obtained from livers of 6 females and 6 males of different litter per group; *p≤0.05.

Because GR expression may be regulated by epigenetic mechanisms like DNA methylation, we studied whether changes in the GR expression were related to altered GR promoter methylation. Several GR mRNA transcripts have been detected in the rat with eleven different exon 1 sequences whose abundance is highly depends upon the tissue [17]. Since exon 1_10_ is highly expressed in the rat hepatic tissue we studied the methylation of 9 CpG sites along its sequence [17]. Fig. 2 A shows the relative positions of alternative exons 1 of the rat GR gene, and Fig. 2 B and C show the methylation of the entire sequence (−2536 to −2361) in liver of female (2 B) and male (2 C) offspring. Differential methylation is observed across groups (control vs Cd -treated) and sex (male vs female) offspring at all CpG sites in the GR1_10_. In female offspring of exposed dams (FT, Fig. 2 B), the methylation status in all the 9 CpG sites of the hepatic GR promoter is significantly lower compared to controls (FC, 62±11%, p<0.05; Fig. 2 B), with statistical significance at sites 1 and 6. In an opposite manner, in treated males (MT, Fig. 2 C) all the 9 CpG sites are hypermethylated compared to respective control (MC,185±21%, p<0.001), with significant differences at sites 1, 6, 7 and 9. Table 1 shows the percentage of GR1_10_ promoter methylation –GR expression related to respective control group.

**Table 1:** Liver GR1_10_ promoter methylation and expression -relative to the control group - of offspring exposed to Cd during gestation.

		Control	Cd-treated during gestation	*p*
GR methylation (all CpG sites)	Female	100	62.2±11	*p<*0.05
	Male	100	185±21	*p*<0.001
GR expression	Female	100	178±7	*p*< 0.004
	Male	100	56±9	*p*< 0.004

### Expression of Methylation Enzymes DNMTs (DNMT1a and DNMT3a)

**Figure 3:**
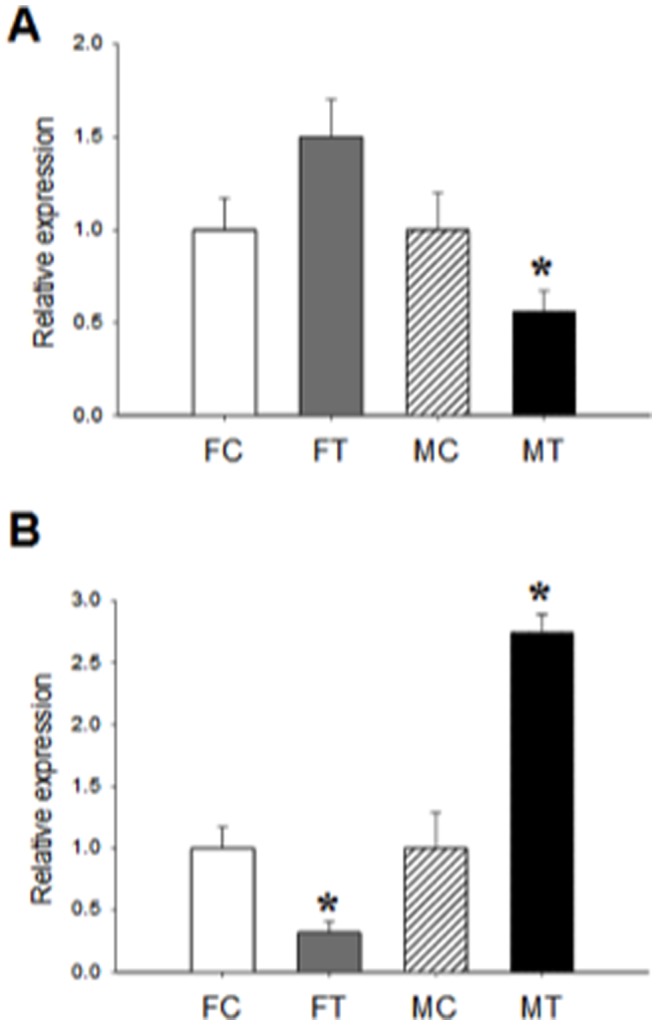
Expression of DNMTs in fetal liver. DNMT1a mRNA was determined by real time PCR as described in Material and Methods (A). Results represent the DNMT1a/β actin ratio of duplicate experimental determination of 6 different biological samples of female control (FC) and Cd-treated offspring (FT) and male control (MC) and Cd-treated offspring (MT). DNMT3a mRNA was determined by real time PCR as described in Material and Methods (B). Results are the DNMT3a/β actin ratio of duplicate experimental determination of 6 different biological samples per group; *p<0.05 compared with respective control.

DNA methylation involves the transfer of a methyl group from the methyl donor SAM to form 5-mC under the catalytic enzymatic effect of DNMTs [32]. DNMT1a is involved in maintenance methylation of the nascent DNA strand following replication and, DNMT3a is involved in the *de novo* methylation as essential mechanisms for vertebrate embryonic development [33]. To study whether the observed changes in GR1_10_ methylation were related with altered expression of DNMTs, we studied the mRNA of both isoforms. As shown in Fig. 3 A, DNMT1a does not change in treated females (FT compared to FC) but decrease in males (MT compared to MC) showing no relation to the effects found in the methylation status of GR1_10_. As opposed, the mRNA expression of DNMT3a (Fig. 3 B) fully reflects the methylation status of GR promoter: in treated females (FT) the mRNA is lower than the control (FC), explaining the hypomethylation observed in GR1_10_. On the contrary, in Cd-treated male fetuses (MT), DNMT3a mRNA increases more than 2 fold compared to controls (MC), mirroring the hypermethylated effect of prenatal exposure to Cd in male liver GR1_10_ promoter.

### SAM and SAH Determination

To study whether methylation changes observed in GR1_10_ were due to differences in the liver concentration of the methylation substrate SAM, we determined its concentration in fetal liver. As shown in Table 2, no differences in SAM or SAH concentrations and in the SAM/SAH ratio were detected in fetal liver of both groups.

**Table 2:** SAM and SAH concentration in fetal liver.

	Control	Cd-treated during gestation
SAM (nmol/g wet tissue)	63.9±11	61±10
SAH (nmol/g wet tissue)	3.2±0.3	2.3±0.6
SAM/SAH	22±3	28±6

### Expression of PEPCK and AOX (mRNA and Protein) in Fetal Liver

**Figure 4:**
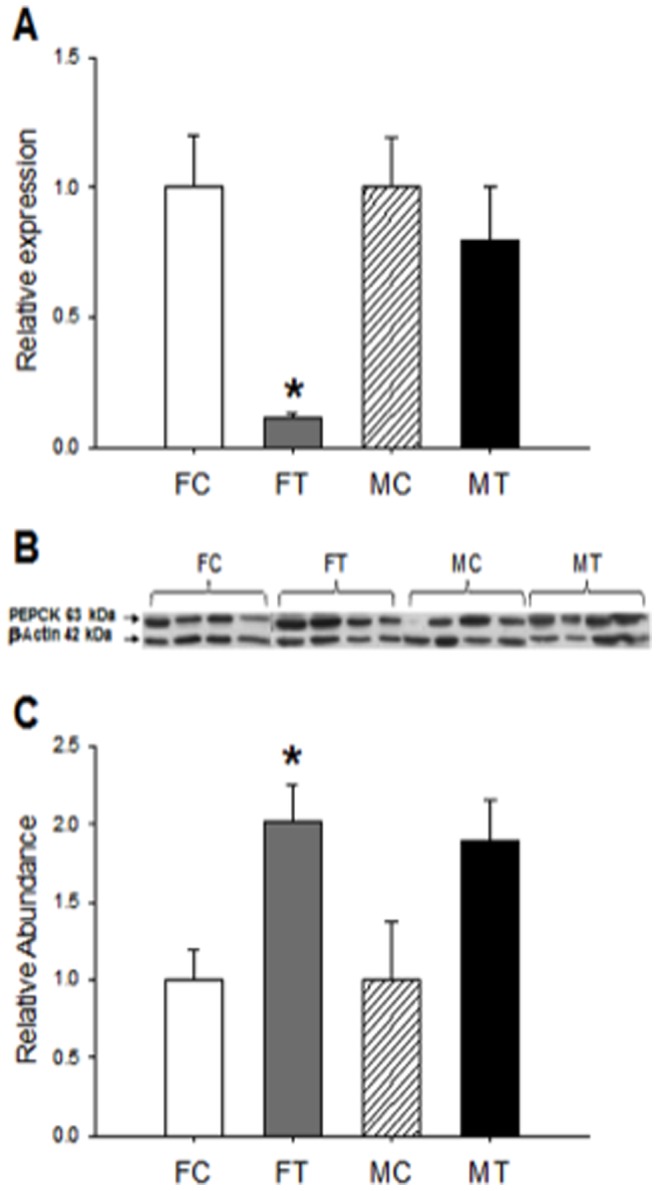
Expression of PEPCK in fetal liver. PEPCK mRNA was determined by real time PCR as described in Material and Methods (**A**)**.** Results represent the PEPCK/β actin ratio of duplicate experimental determination of 6 different biological samples of female control (FC) and Cd-treated offspring (FT) and male control (MC) and Cd-treated offspring (MT). Western blot analysis for PEPCK protein expression in liver of control (FC) and treated female (FT) and control (MC) and treated male (MT) fetus (B). PEPCK western blot signals were determined by densitometry and expressed as abundance ratios of PEPCK related to actin (C); *p<0.05 compared with respective control.

To examine whether changes detected in fetal liver GR affect some of their target genes involved in hepatic metabolism we examined the expression of PEPCK and AOX. Results of PEPCK in Fig. 4 show that in exposed females (FT), PEPCK mRNA is lower than controls (FC, Fig. 4 A) and the protein is more abundant than the controls (Fig. 4 B and C), suggesting that in females the GR target gene is differentially regulated at transcription and traduction levels by prenatal exposure to Cd. On the contrary, no differences are observed in males by the prenatal exposure to Cd (MT vs MC), and in consequence, the mRNA of PEPCK as well as the protein remained unmodified by the treatment (Fig. 4 A, B and C).

**Figure 5:**
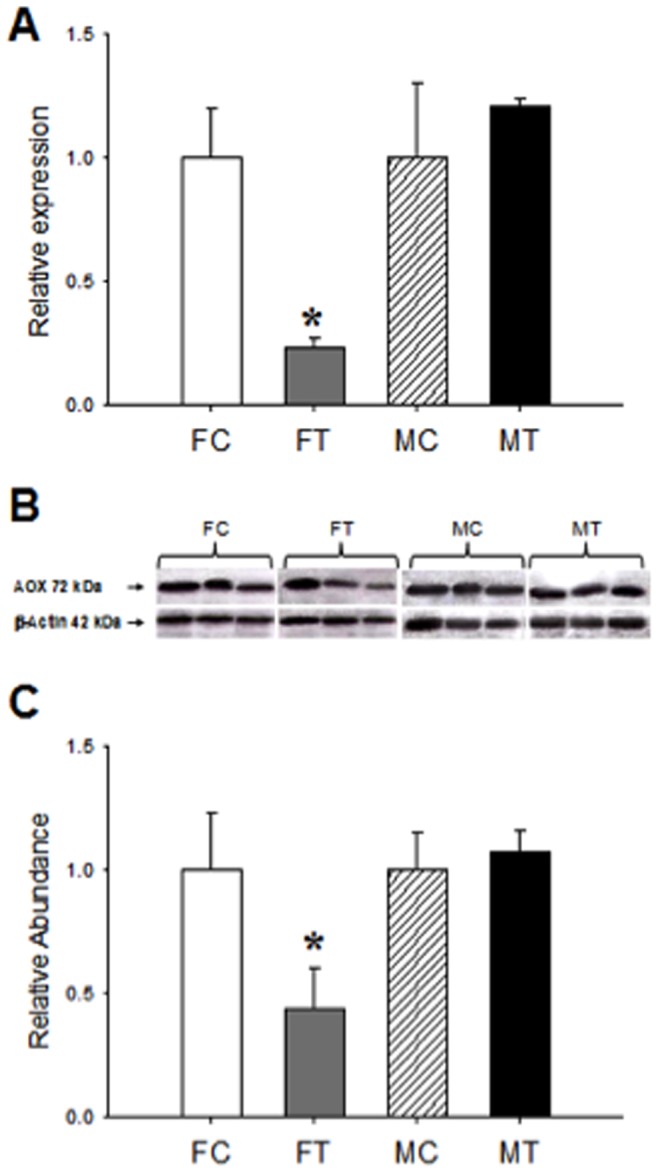
Expression of AOX in fetal liver. AOX mRNA was determined by real time PCR as described in Material and Methods (A). Results represent the AOX/β actin ratio of duplicate experimental determination of 6 different biological samples of female control (FC) and Cd-treated offspring (FT) and male control (MC) and Cd-treated offspring (MT). Western blot analysis for AOX protein expression in liver of control (FC) and treated female (FT) and control (MC) and treated male (MT) fetus (B). AOX western blot signals were determined by densitometry and expressed as abundance ratios of AOX related to actin (C); *p<0.05 compared with respective control.

Results of AOX expression (mRNA and protein) in liver of fetal females and males are shown in Fig. 5. In exposed females (FT), AOX expression (mRNA and protein) is lower than the control (FC). As opposed, in males, no differences are observed by the prenatal exposure to Cd (MT vs MC), and in consequence, the mRNA of AOX as well as the protein were unmodified by the treatment.

## Discussion

In this study we provide evidence that Cd exposure to pregnant rats (50 ppm), affects birth and size of the offspring, suggesting that this toxic metal, at the concentration used in this study, alters the placental environment and may induce developmental programming. Developmental alterations induced by Cd include changes in the methylation and expression of the fetal liver GR in a sex-dependent manner. The changes in GR methylation were highly correlated with changes in DNMT3a expression. This observation suggests that prenatal exposure to Cd affects the *de novo* methylation reactions in fetal liver resulting in changes of GR1_10_ methylation. Importantly, the alterations in the expression of fetal liver GR and DNMT3a by Cd treatment were sex-dependent, in an opposite manner. Although these changes do not prove causality with the lower fetal growth, which was observed in both, males and females, they may induce important long-term effects on health. The modified epigenetic state of the fetal liver GR gene in response to prenatal exposure to Cd is noteworthy as it implies an effect of maternal Cd intake on programming the hypothalamus-pituitary-adrenal (HPA) axis as previously suggested by us [10].

The results of this study led us to hypothesize that Cd may act as an endocrine disruptor on the GC system involving specific changes in the GR through alterations in the DNA methylation of its promoter sequence Only in female neonates, the hepatic GR1_10_ expression was related to the expression of PEPCK and AOX, two enzymes associated with the carbohydrates and lipids metabolism respectively.

It is important to note that the methylation status of the fetal liver GR1_10_ promoter in a basal state was low, and, although the prenatal Cd treatment induced changes at the average CpG methylation status, these modifications were slight. When examined individually, the 9 CpG sites of the GR1_10_ sequence of treated female fetuses were less methylated than their controls; the opposite was found in male fetuses. However, when examined individually, only few CpG units were significantly different in the treated fetuses compared to controls. Although the significance of relatively small changes in DNA methylation is unclear, such modest differences in methylation in some CpG islands have been documented in human studies in intrauterine growth restricted neonates (IUGR), and consequently, small changes may affect fetal growth [34]. These authors focused their studies in the DNA methylation of stem cell populations of the developing fetus and demonstrated that epimutations accumulate over time being mitotically heritable and could be potentially transmitted to the next generation [34], [35].

In human placenta, Filiberto et al., 2011 [36] found a significant association between differential methylation at exon 1F of the GR gene and large for gestational age (LGA) status, suggesting a link between infant growth and epigenetic alteration of GR. A recent study in humans, reported a higher average CpG methylation of a cortisol-regulating gene promoter by a higher methyl donor intake, without significant differences in each individual CpG unit of the analyzed sequence [37].

We have previously demonstrated that the same level of Cd (50 ppm) exposure to pregnant mothers did not reach fetal tissues [38]. This observation suggests that the effect on fetal liver GR may be a consequence of a placental dysfunction due to Cd accumulation, affecting the development of the fetus.

Environmental chemicals may modify multiple biological processes that affect epigenetic mechanisms, including DNA methylation, histone codes, and miRNA expression [39], [40]. These modifications may affect chromatin organization and condensation, gene expression, and increase the risk of a disease [41]. Epigenetic modifications of gene expression, mainly related to promoter methylation, have been suggested as an important molecular mechanism involved in environmental programming.

Periconceptional exposure to environmental factors, such as heavy metals, is known to cause epigenetic alterations that persist throughout the life of higher organisms [42], [43]. Specifically, it has been reported that some of the toxic effects of Cd are mediated by epigenetic mechanisms, like DNA methylation, through an alteration of DNMTs expression [25], [40], [44], [45]. Previous *in vitro* studies have demonstrated that Cd is able to alter total DNMTs activities [25]; and more specifically, DNMT1, 3a and 3b mRNAs were up-regulated by long-term Cd exposure [44]. In an embryo chick model, Cd exposure induced altered expression of the DNMT3A/3B genes resulting in global DNA methylation changes during critical periods of early chick embryogenesis [32], [33], [45]. In our study, prenatal Cd exposure altered, in a sex-dependent manner, the expression of the fetal liver *de novo* methylation gene DNMT3A, suggesting that this may be the cause of the GR1_10_ methylation changes. These results are the first demonstration of a sex-dependent effect on hepatic DNMT3a by prenatal exposure to Cd in rats.

Altered methylation of the hepatic GR promoter (at GR1_10_) has been linked to prenatal protein restricted diets; and was correlated to altered DNMT1 expression, suggesting that different adverse signals during fetal development can alter epigenetic marks in a different way [21]. Unfortunately, the authors of the later study did not specify the sex of the offspring in which gene expression measurements were performed. Genomic methylation pattern are acquired in the germ line and differ markedly for male and female gametes [46]. Also, DNMT3a displays unique developmental profiles that show marked sex-specific differences [47]. In this respect, it has been described that some environmental factors during gestation can affect differentially female and male offspring, inducing permanent gender-specific differences in physiology and cardiovascular disease risk at adult age [48], [49]. Gender differences have been observed by GC exposure during late gestation in rats, and also in humans, where an association between maternal Cd exposure and birth size was apparent only in girls [50], [51].

The expression of GR was inversely associated with the methylation status, then, GR of treated females was more expressed than controls, and GR of males presented lower expression. In general, a hypermethylated gene at its GpG promoteŕs region is more expressed than a hypomethylated gene [52], [53].

The pattern of expression of GR mRNA found in our study was also detected at the GR protein level, and then, exposed females expressed more GR protein than controls and males expressed less GR protein than their respective controls (Fig. 1).

Since in this study males and females fetuses exposed to Cd during gestation showed lower birth weight than the controls, it is difficult to interpret the birth weight in terms of a greater or lesser liver GR expression at birth. A recent study showed that IUGR induces an increase of the CpG methylation status, as well as changes in the pattern of methylation in several CpG sites of 11β-HSD2 promoters in the kidney of rat neonates in a sex-specific manner suggesting a link between fetal growth and risk of hypertension at later age [49].

The interaction of some environmental chemicals with sex-steroid hormone responses has been well documented. These “endocrine disruptors” interfere mainly with mechanisms regulated by sex steroid hormone receptors affecting sex steroid hormone action [54]. This effect has been described for some heavy metals and, specifically, for Cd [55]–[57]. It has been suggested that Cd interacts with the estrogen receptor alpha (ER) through an interaction with the hormone-binding domain of the receptor, which binds with high affinity, blocking the binding of estradiol [55], [56]. Deregulation of ER activity by environmental chemicals has been suggested to be involved in the development of reproductive disorders, however, disturbances of ER-mediated processes and Cd-mediated effects on progesterone synthesis in ovary and placenta cannot explain the complex toxic effects of most of the compounds disrupting endocrine regulation [12], [58], [59]. An effect of environmental chemicals on the GC system has also been reported, and results of the present study may give support to a potential endocrine disruptor effect of Cd on the GC system during development [60]. First potential endocrine effects of Cd on the GC system were described by Yang et al, (2006) who reported inhibitory effects of Cd on 11β-HSD2 in cultured human trophoblast cells [24]. More recently, we reported increased levels of maternal and fetal corticosterone by Cd exposure during pregnancy [10]. This result may be a consequence of an endocrine disruptor effect of Cd on the HPA axis during development, suggesting that adrenal activation is dependent on the presence of the metal in the blood [61]. Alternatively, it may be related to a Cd effect on the 11βHSD-2 at placental level. A fetal overexposure to maternal GC during development may decrease offspring birth weight and permanently alter the HPA axis with deleterious long time consequences.

In summary, this study demonstrates for the first time that, exposure to Cd during gestation may alter the *de novo* DNA methylation in fetal liver of rats and, consequently, the methylation status of GR with gender-specificity. It remains to be determined whether the effects on GR gene methylation and expression persist throughout the lifespan of the offspring, and whether the hepatic metabolism of carbohydrates and lipids may be altered at adult age as a consequence of a GR disruption induced by prenatal exposure to Cd.

## Materials and Methods

### Animals and Treatments

**Figure 6:**
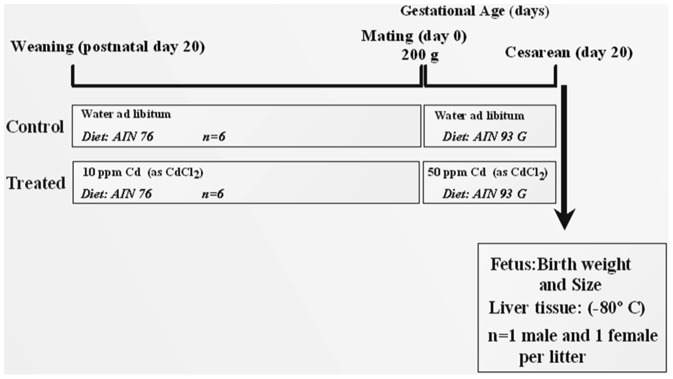
Schematic diagram of the experimental protocol. The sequence of experimental procedure and number of dams and fetuses involved in each stage of the study.

Female Wistar rats were fed with AIN-76 diet and had drinking water with (exposed) or without (control) 10 ppm of Cd (as CdCl_2_) *ad libitum* from weaning to mating at 200 g of weight. Mating was confirmed by the presence of a vaginal copulation plug (day 0 of gestation) when they were divided in two groups: (a) control (n = 6), feed with diet (AIN-93G) and distilled drinking water *ad libitum*; and, (b) exposed (n = 6), feed with diet AIN-93G and 50 ppm of Cd (CdCl_2_ in the distilled drinking water) *ad libitum*. Pregnant rats (n = 12) were individually housed and maintained at 12 h light: 12 h dark schedule. Treatments continued during the whole pregnancy period until 1 day before delivery (day 20 of pregnancy). All procedures were performed according to the guidelines of the American Veterinary Medical Association (AVMA) (Report of the AVMA, Panel on Euthanasia, 2001) [62] and approved by our local Bioethics Committee for Animal Experimentation at the Institute of Nutrition and Food Technology (INTA), University of Chile, Santiago, Chile. The schematic diagram of the experimental protocol is depicted in Fig. 6.

### Measurements and Sample Extraction

Maternal weight, water and food consumption were monitored throughout gestation. At day 20 of gestation, pups were delivered by caesarean section. The birth weight and size of males and females offspring was registered and the liver was collected, weighed, and frozen at −80°C for subsequent molecular analysis. For methylation and gene expression analysis, livers from one female and one male per litter were randomly chosen (n = 6 per group) and processed.

### Analysis of the Methylation Status of Individual CpG Dinucleotide in Hepatic GR1_10_ Sequence

DNA was extracted from liver neonates by Wizard genomic DNA purification kit (Promega) and treated with sodium bisulfite using the EZ DNA Methylation-Gold kit (Zymo Research) according to the manufacturer’s instructions. The bisulfite reaction was performed on 0.5 µg DNA and the reaction volume was adjusted to 20 µl with sterile water and 130 µl of CT conversion reagent was added. PCR was conducted with biotinylated primers (Assay ADS086, designed by EpigenDx, USA). The PCR products (10 µl each) were sequenced by pyrosequencing PSQ96 HS System (Biotage), a real-time sequencing-based DNA analysis that quantify multiple and consecutive CpG sites individually. At first, the methylation assay covered 30 CG dinucleotides in promoter region ranging from −2536 to −2361 in reference to transcriptional start site. Afterward, a nested PCR covering 9 CpG island was performed (ADS 086FS2). The methylation status of each locus was analyzed individually as a T/C SNP using QCpG software (Biotage). PCR cycling conditions were: 94°C 15 min; 45 cycles of 94°C 30 s; 59°C 30 s; 72°C 30 s; 72°C 5 min.

### Determination of GR, PEPCK, AOX and DNMTs (1 and 3a) mRNA in Fetal Liver by qRT-PCR

The expression of mRNA for all genes was quantified by quantitative real time PCR. Total RNA was extracted from 50 mg of liver of male and female neonates using TRI Reagent kit (Ambion). Actin was used as housekeeping gene. The gene transcript levels were quantified separately using the LightCycler® FastStart DNA Master SYBR Green I kit (Roche) and the following programs for specific genes were applied: GR, activation step at 95°C for 10 min followed by an amplification step with 40 cycles of 94°C 5 s, 56°C 6 s and 72°C 10 s; PEPCK, activation step at 95°C for 10 min followed by an amplification step with 40 cycles of 94°C 5 s, 62°C 10 s and 72°C 6 s; AOX, activation step at 95°C for 10 min followed by an amplification step with 40 cycles of 94°C 5 s, 62°C 10 s and 72°C 7 s; DNMT1a, activation step at 95°C for 10 min followed by an amplification step with 40 cycles of 94°C 5 s, 60°C 6 s and 72°C 12 s; DNMT3a, activation step at 95°C for 10 min followed by an amplification step with 40 cycles of 94°C 5 s, 62°C 5 s and 72°C 14 s; and β-actin, activation step at 95°C for 10 min followed by an amplification step with 40 cycles of 94°C 5 s, 59°C 6 s and 72°C 20 s. The mRNA was quantified by the relative standard curve method, and the relative amount of the respective mRNAs was normalized to the values of β-actin. Primer sequences of all genes are described in Table 3. Data represent the averaged of two experimental replicates from liver samples of 6 males and 6 females per group.

**Table 3:** Rat SYBR Green I assay primers.

Name	Sequence	Nucleotide location
*GR fw*	5′-CCTCCCATTCTAACCATCCT-3′	4244–4263
*GR rv*	5′-CTCCCTCTGCTAACCTGTG-3′	4400–4418
*PEPCK1* fw	5′-ACGGTGGGAACTCACTGCTTG-3′	820–840
*PEPCK1* rv	5′-TGCCTTCGGGGTTAGTTATGC-3′	922–942
*AOX1 fw*	5′-ACAAGCTGACGTATGGGACC-3′	902–921
*AOX1 rev*	5′-GTGGTTCTGGTTCGCTTTGC–3′	1029–1048
*DNMT1 fw*	5′-CGTGACCTGCCCAACATACA-3′	4367–4386
*DNMT1 rv*	5′-GAAGAAGCCATCCCACTCCA-3′	4605–4624
*DNMT3a fw*	5′-TGTACCGCAAAGCCATCTACG-´3	568–588
*DNMT3a rv*	5′-CTCATACACGAGCCGTTCTC-´3	904–923
*β-actin fw*	5′-CCGTAAAGACCTCTATGCCA-3′	948–967
*β-actin rv*	5′-AAGAAAGGGTGTAAAACGCA-3	1227–1246

Nucleotide locations are derived from published *Rattus norvergicus*: GR mRNA (GenBank acc. no NM_012576), Phosphoenolpyruvate Carboxykinase 1 (PEPCK1) mRNA (GenBank acc. BC081900), Acyl- CoA oxidase 1 (AOX1) mRNA (GenBank acc. NM_017340), DNA methyltransferase (cytosine-5) 1 (Dnmt1) mRNA (GenBank acc. no NM_053354), DNA methyltransferase 3 (Dnmt3a) mRNA (GenBank acc. no NM_001003958) and β-actin (Actb) mRNA (GenBank acc. no NM_031144).

### Determination of GR, PEPCK and AOX by Western Blot

Aliquots of homogenized liver were separated using 10% polyacrylamide gel (PAGE) containing sodium dodecyl sulfate (SDS) along with a pre-stained, broad-range, molecular weight marker (New England Biolabs). The separated proteins were transferred onto polyvinylidene fluoride (PVDF) membranes (Thermo Scientific, Rockford, USA) overnight at 4°C. The membranes were then blocked with 5% non-fat milk in Tris buffered saline with 1% Tween-20 (TBS-T) for 1 h and then incubated with a specific primary antibody against GR (Abcam) at 5 µg/ml or against PEPCK (Santa Cruz Biotechnology) at 1 µg/ml and against AOX1 (Santa Cruz Biotechnology) at 2 µ/ml. For internal control, membranes were incubated with primary antibody against β-actin (Abcam) at a dilution of 1∶7,000. For GR, the membranes were washed with TBS-T and incubated for 1 h with goat anti-mouse secondary antibody–horseradish peroxidase conjugates (Chemicon) at a 1∶1,500 dilution. PEPCK1 and AOX1 membranes were washed and incubated for 1 h with goat anti-rabbit secondary antibody-horseradish peroxidase conjugates (Santa Cruz) at a 1∶2,000 dilution. Specific bands were developed using an enhanced chemiluminescence (ECL) Western blotting detection kit (Lightning Plus-ECL, Perkin Elmer) and exposing the membranes to film (Amersham) to obtain a signal. Bands were measured using densitometry with software (Image J).

### Hepatic SAM and SAH Determination

SAM and SAH concentrations in liver homogenates were quantified by HPLC by using an Agilent-1100 DAD detector (Hewlett Packard) operating at 260 nm as described [63]. Frozen liver was weighed, homogenized with HClO_4_ 0.5 M 1∶5 (w/v) in ultraturrax (Heidolph Diax 900) and centrifuged at 12,500×*g* for 5 min. Supernatant was filtered through a 0.22 µm Millipore filter. Acid filtrates were directly injected to the HPLC (25 µl). A Hypersil BDS column C18 (53×7.0 mm, 3 µm, Alltech Rocket, PA, USA) was used, with a mobile phase that consisted of 40 mM NH_4_H_2_PO_4_, 8 mM 1-heptanesulfonic acid, and 18% (v/v) methanol, pH adjusted to 3.0 with HCl. Under these conditions, retention times for SAH and SAM were 3.3 and 4.4 min respectively. HPLC analyses were conducted at a flow rate of 3 ml/min at 35°C. Calibration curves were based on peak area, and linear response was obtained between 10 and 1000 pmol for SAM or SAH (Sigma) with a correlation coefficient greater than 0.999 for each curve. The concentrations of SAM and SAH related linearly to the areas under the HPLC chromatogram. Results were expressed as nmol per g of wet tissue.

### Statistical Analyses

Analysis of sample size was performed using Primer for Biostatistics Program by Stanton A. Glantz 3.2, 1992. Considering a difference in GR expression of 0.3 units (normalized to β-actin expression), a SD of 0.1, an α error of 0.05 and a power of 0.8, the sample size was 4 per group. We added an insurance margin of 50% and increased the n to 6 animals per group. For statistical analysis the SPSS and Prisma program were used. For fetal characteristics, the Shapiro-Wilk test was used to evaluate normality of data. When data were normally distributed, results were expressed as means±SD; when data were abnormally distributed, data were expressed as medians and percentiles (p25–p75). The non-parametric Kruskal-Wallis and Mann-Whitney tests were used to compare differences among groups and between two groups respectively. Values representing methylation of individual CpG dinucleotides of each group were expressed in percentage. Differences were considered statistically significant at p<0.05.
